# Imaging-Based Clinical Management of Mandibular Canal Variants: PR–CBCT–Selective MRI

**DOI:** 10.3390/biomedicines13112760

**Published:** 2025-11-12

**Authors:** Ingrid C. Landfald, Magdalena Łapot, Łukasz Olewnik

**Affiliations:** 1 Department of Clinical Anatomy, Mazovian Academy, Plac Dąbrowskiego 2, 09-402 Płock, Poland; i.landfald@mazowiecka.edu.pl (I.C.L.); m.lapot@mazowiecka.edu.pl (M.Ł.); 2 VARIANTIS Research Laboratory, Mazovian Academy, Plac Dąbrowskiego 2, 09-402 Płock, Poland

**Keywords:** mandibular canal, inferior alveolar nerve, anterior loop, bifid canal, retromolar canal, CBCT, MRI, anesthesia, dental implants, third-molar surgery

## Abstract

**Background:** Mandibular canal (MC) variants are common and clinically relevant for anesthesia, implant placement, third-molar surgery, and osteotomies. Reported prevalences vary widely because they depend on imaging modality, acquisition parameters, and operational definitions. **Methods:** This was a focused narrative review with structured methods (PubMed/MEDLINE and Scopus, 2000–6 October 2025; last search 6 October 2025), predefined eligibility criteria and dual independent screening; no meta-analysis was conducted. Study-selection counts are reported in the text. Prevalence statements are contextualized by modality, imaging parameters (e.g., cone-beam computed tomography (CBCT) voxel size magnetic resonance imaging (MRI) field strength/sequences), and diagnostic thresholds (e.g., anterior loop (AL) criteria). **Results:** Compared with panoramic radiography (PR), CBCT consistently reveals more variant pathways. Typical CBCT estimates for bifid MC fall in the single-digit to low double-digit range, contingent on voxel size and definitions, whereas PR detects far fewer. Trifid canals are uncommon (≈1–2% in CBCT series). Reported retromolar canal frequencies vary broadly across populations and protocols, and AL length and prevalence are threshold-dependent. Selective MRI may complement CBCT by depicting soft-tissue branches not accompanied by a bony canal. We synthesize a variant-aware, imaging-led workflow: PR for screening; CBCT when predefined criteria are met and results are reasonably expected to change management; MRI reserved for targeted soft-tissue questions, in line with As Low as Reasonably Achievable (ALARA)/and As Low As Diagnostically Acceptable (ALADA) principles. We apply the Landfald Clinical Framework (LCF) as a hypothesis-generating, clinical synthesis tool linking variant patterns to procedural modifications and risk mitigation. **Conclusions:** A narrowed, clinically oriented approach—contextualizing prevalence by modality and definitions and applying an imaging-led, variant-aware workflow—can improve planning and safety in the posterior mandible. The LCF is used pragmatically within this workflow and does not constitute a new anatomical taxonomy; formal reliability and validity testing remain necessary.

## 1. Introduction

The mandibular canal (MC) and inferior alveolar nerve (IAN) are critical anatomical structures encountered in a wide range of oral and maxillofacial surgical procedures. The MC originates at the mandibular foramen (MF) on the medial surface of the mandibular ramus and extends anteriorly to terminate at the mental foramen (MeF). It contains the inferior alveolar neurovascular bundle, which includes the IAN, inferior alveolar artery (IAA), and vein, providing innervation and vascular supply to the mandibular dentition, buccal gingiva, and lower lip [1,2].

Historically, limited awareness of anatomical variability within the MC led to a widespread assumption of structural uniformity. Clinical practice relied heavily on two-dimensional panoramic radiography (PR), which frequently failed to identify canal variants due to image distortion, structural superimposition, and poor spatial resolution [3,4,5,6]. The advent of cone-beam computed tomography (CBCT) and magnetic resonance imaging (MRI) has significantly improved the visualization of the MC, revealing substantial variation in its trajectory, branching patterns, and vertical position [3,7,8]. This technological shift has prompted a paradigm change in surgical planning away from standardized approaches and toward individualized, variant-aware strategies [9].

Several variant configurations, including bifid and trifid canals, retromolar canals, anterior loops (AL), and accessory foramina, have been increasingly reported in the literature and are now considered common rather than exceptional anatomical findings [10,11,12]. These variations may impact procedural accuracy and are relevant for local anesthesia efficacy, bleeding risk, and postoperative neurosensory outcomes [7,11].

The aim of this review is to consolidate anatomical, radiological, and developmental data regarding MC and IAN variability and to examine the implications of such findings in the context of modern surgical practice. A classification-based framework is also proposed to facilitate systematic identification and documentation of MC variants. This is not a proposal of a new anatomical taxonomy; we apply an established classification in a pragmatic, imaging-led clinical workflow. This review delivers a focused, clinically oriented synthesis of MC variants that directly informs surgical planning in the posterior mandible. We outline a practical, variant-aware imaging workflow that begins with PR for screening, uses CBCT when predefined criteria are met, and reserves MRI for selected soft-tissue questions, in line with As Low As Reasonably Achievable (ALARA) and As Low As Diagnostically Acceptable (ALADA) principles. Prevalence data are contextualized by imaging modality and operational definitions (e.g., voxel size; AL thresholds), and implications are summarized for anesthesia, implant positioning, third-molar surgery, and osteotomies. We present the Landfald Clinical Framework (LCF) as a hypothesis-generating tool linking variant patterns to procedural modifications; formal reliability and validity testing remain necessary. Accordingly, the framework is used as a clinical synthesis tool rather than an anatomical reclassification Detailed step-by-step operative techniques and broad educational content are intentionally outside the scope of this article. We retained a single integrated review rather than split the content into separate articles because chairside decisions depend on the joint consideration of variant anatomy, modality choice, and procedural risk. To preserve clinical focus, we trimmed didactic/operative detail and emphasized imaging-led decision points.

## 2. Materials and Methods

### 2.1. Review Design and Protocol

We conducted a focused narrative review using structured methods to ensure transparency, traceability, and basic reproducibility appropriate for narrative reviews. Reporting follows PRISMA 2020 [13] items where applicable to qualitative syntheses; no meta-analysis was planned. Predefined eligibility criteria, dual independent screening, standardized data extraction, and qualitative risk-of-bias considerations (referencing the JBI checklist for prevalence studies) are detailed in Section 2.3, Section 2.4, Section 2.5, Section 2.6 and Section 2.7. Information sources and search dates are provided in Section 2.2. Searches were limited to English. No prospective protocol was registered.

### 2.2. Information Sources and Search Strategy

We searched PubMed/MEDLINE and Scopus for human studies published from 1 January 2000 to 6 October 2025 (last search: 6 October 2025). Searches combined controlled vocabulary and free-text terms related to the MC, the inferior alveolar neurovascular bundle, and imaging modalities of interest (CBCT, MRI). Full, exact search strings and limits for both databases are provided in Table S1 (Supplementary).

PubMed (example search string):

(mandibular canal[Title/Abstract] OR “inferior alveolar nerve”[Title/Abstract] OR “bifid mandibular canal”[Title/Abstract] OR retromolar canal[Title/Abstract] OR “anterior loop”[Title/Abstract] OR “accessory mental foramen”[Title/Abstract]) AND (“cone-beam”[Title/Abstract] OR CBCT[Title/Abstract] OR “magnetic resonance”[Title/Abstract] OR MRI[Title/Abstract]) AND (humans[MeSH Terms] OR humans[Title/Abstract]).

Scopus (example search string):

TITLE-ABS-KEY(mandibular W/1 canal OR “inferior alveolar nerve” OR “bifid mandibular canal” OR “retromolar canal” OR “anterior loop” OR “accessory mental foramen”) AND TITLE-ABS-KEY(“cone-beam” OR CBCT OR “magnetic resonance” OR MRI) AND DOCTYPE(ar OR re) AND LANGUAGE(english) AND PUBYEAR > 1999.

Reference lists of included articles and key reviews were scanned to identify additional relevant studies.

**Figure 1:**
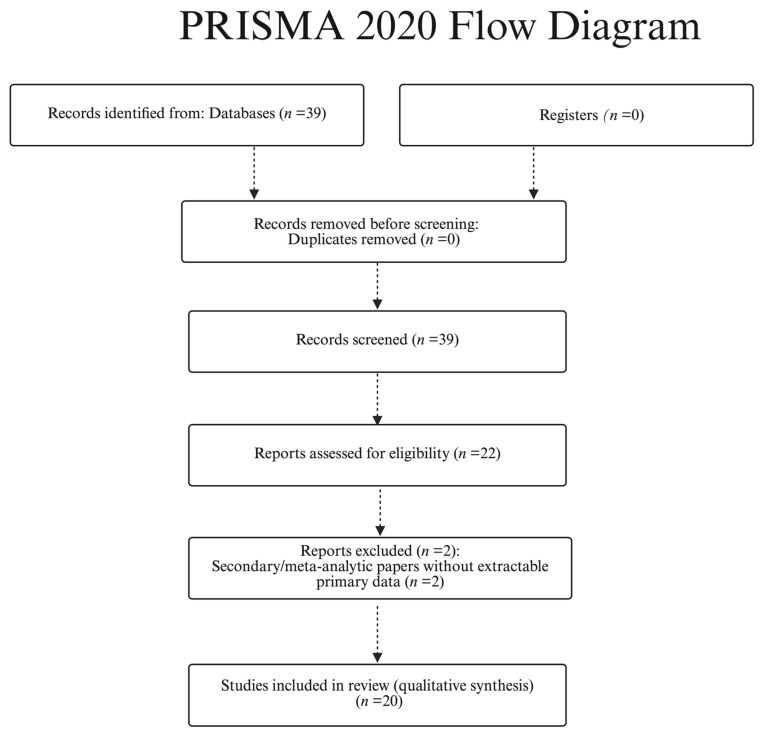
PRISMA 2020 flow diagram for study selection (PubMed/MEDLINE and Scopus; last search: 6 October 2025).

Study selection (PRISMA-style): We identified 39 records from PubMed/MEDLINE and Scopus (last search 6 October 2025). No duplicates were found; therefore, 39 titles/abstracts were screened and 22 full texts were assessed for eligibility. 20 studies were included in the qualitative synthesis (Table 1). Full-text exclusions (*n* = 2) were secondary/meta-analytic papers without extractable primary data; title/abstract exclusions (*n* = 17) were pediatric-only cohorts without adult strata, case reports without generalizable measurements, or articles with unclear imaging parameters. See Figure 1 (PRISMA flow diagram).

### 2.3. Eligibility Criteria

**Inclusion criteria**

- Human studies (imaging or anatomical) reporting prevalence or measurable features of MC-related variants (e.g., bifid/trifid canals, retromolar canal (RMC), AL presence/length, AMF, high-positioned canal).
- Imaging context explicitly stated, including at minimum modality and acquisition parameters (e.g., CBCT voxel size; for MRI, field strength and sequences).
- Original data (observational cohorts, cadaveric series, imaging audits), or systematic reviews with extractable primary data (used cautiously, avoiding double-counting).

**Exclusion criteria**

- Non-human studies; pediatric-only cohorts without separate adult reporting; case reports without generalizable measurements; conference abstracts without methods; duplicate cohorts (the most comprehensive/recent report retained).

Language limits are stated in Section 2.1.

### 2.4. Study Selection

Two reviewers independently screened titles/abstracts and then full texts against the eligibility criteria. Disagreements were resolved by consensus. When necessary, uncertainty about duplicate cohorts or overlapping recruitment periods was resolved by preferring the study with larger sample size, clearer imaging parameters, and/or more complete reporting.

### 2.5. Data Extraction

A standardized extraction form captured the following:

- Study identifiers: first author, year, country, design, sample size, population characteristics.
- Imaging details: modality (PR/CBCT/MRI), CBCT voxel size and reconstruction parameters; MRI field strength and sequences (e.g., T1/T2, DESS, DTI where applicable).
- Operational definitions/thresholds: criteria used to define each variant (e.g., definition of bifid canal; AL measurement method and threshold), coordinate planes used for measurements.
- Outcomes: prevalence (%) and/or morphometrics (e.g., AL length), inter-/intra-observer agreement if reported, and reported clinical implications (anesthesia failure, implant safety margins, third-molar risk, osteotomy planning).

Extraction was performed independently by two reviewers; discrepancies were reconciled by discussion, consulting the full text and figures as needed.

### 2.6. Outcomes and Definitions (A Priori)

Primary outcomes: prevalence of MC variants (BMC/TMC, RMC/RMF, AL, AMF, high-positioned canal). Secondary outcomes: AL length, MC-to-crest height (mm), inter-/intra-observer agreement. Operational definitions/thresholds are consolidated in Box 1 and applied in Table 1 (per-study) and Table 2 (decision keys); Section 7.1 lists point-of-use thresholds. Strata by voxel size/MRI sequence were extracted separately.

Box 1 **Operational definitions & thresholds (applied across Table 1 and Table 2).**

General confirmation rules (CBCT).

- A variant is present when a corticated tract/foramen is visible in ≥2 orthogonal planes with adequate quality.
- Measurements on MPR with perpendicular calipers; report in mm (1 decimal).
- Discrepancies resolved by consensus between ≥2 readers.

Imaging triggers (selection logic).

- PR for screening → selective CBCT when the result will change management (implant site in variant-rich zones; uncertain mental foramen [MeF] position; suspected anterior loop; retromolar surgery; sagittal split/ramus procedures; discordant PR).
- MRI (3 T) reserved for soft-tissue pathway questions (bundle course; perineural pathology) when CBCT is inconclusive.

Variant-specific definitions.

- BMC/TMC—duplication/splitting of the corticated MC forming ≥2 distinct tracts. Presence: identifiable in ≥2 planes (axial + coronal/sagittal). Report: configuration (bifid/trifid), limb diameter(s), course.
- RMC/RMF—corticated accessory canal to a retromolar foramen posterior to the last molar. Presence: RMF and connecting tract in ≥2 planes. Key measures: MeF–RMF distance; RMF–crest height; canal width.
- AL (IAN)—anterior extension of the IAN beyond the mental foramen (MeF) before looping posteriorly to exit. Presence: pathway consistent with IAN trajectory in ≥2 planes. Measure: loop length (mm) along the neurovascular course.
- HMC—reduced vertical MC–crest clearance at the planned site. Measure: shortest perpendicular distance from MC roof to the alveolar crest in the target region.
- AMF—separate corticated foramen distinct from MeF with an independent tract. Presence: discrete cortical rim and measurable separation from MeF (report bilaterality). Measure: foramen diameter(s), MeF–AMF distance, depth to crest.

Decision thresholds (point-of-use).

- AL: clinically relevant when ≥1–3 mm anterior to MeF (measure along IAN trajectory on MPR in ≥2 planes).
- HMC: MC–crest ≤ 4–5 mm denotes a high-risk corridor at the planned site.
- BMC/TMC & RMC/RMF: limb/tract ≥ ~1 mm or intersecting the planned osteotomy/implant path ⇒ modify plan/re-route.
- AMF: avoid lateral drilling along the AMF axis; margins individualized from CBCT.

Reader methodology & quality control.

- Prefer ≥2 readers; record inter-/intra-observer agreement where available; resolve discrepancies by consensus.

Document voxel size (CBCT) and sequence/field (MRI); minimize metal artifacts; use consistent windowing.

### 2.7. Risk of Bias and Certainty Considerations

Given the observational nature and imaging heterogeneity, we qualitatively assessed risks related to the following: selection bias (clinic- vs. population-based cohorts), imaging bias (voxel size, reconstruction kernels, slice thickness, MRI sequence choice and artifacts), definitional bias (operational thresholds for AL and canal duplication), and measurement bias (lack of blinded dual reading or inter-observer metrics). Findings are synthesized with explicit attention to these sources of heterogeneity; no quantitative pooling was attempted.

### 2.8. Synthesis Methods

Data are presented in comparative tables by variant and stratified by modality and imaging parameters (e.g., voxel size categories; MRI field/sequences). Narrative synthesis highlights how operational definitions and acquisition settings influence the reported ranges. On this basis, we derive a variant-aware imaging workflow (PR for screening; CBCT when predefined criteria are met; MRI reserved for specific soft-tissue questions) aligned with ALARA/ALADA principles.

### 2.9. Deviations from Protocol

No prospective registration was performed. After initial scoping, we narrowed the review to posterior mandibular planning (anesthesia, implants, third molars, osteotomies) and framed the Landfald concept explicitly as a hypothesis-generating framework requiring future validation.

## 3. Results

This section synthesizes findings from included imaging and anatomic studies with attention to heterogeneity in CBCT voxel size, operational definitions (e.g., confirmation in ≥2 planes for bifid canals), and thresholds for the AL. Given these differences, no meta-analysis was performed. Study-level parameters, definitions, and prevalence ranges are compiled in Table 1.

To contextualize prevalence claims without duplicating tabular detail, we summarize ranges by imaging modality, acquisition parameters, and operational definitions; per-study values and criteria appear in Table 1.

**Typical ranges by modality/threshold.** BMC: PR cohorts often report <1% due to superimposition; CBCT typically yields single- to low double-digit values in adult, non-targeted cohorts when voxel size is ~0.2–0.3 mm and confirmation is made in ≥2 orthogonal planes. TMC: ≈1–2% in CBCT series, sensitive to definitional criteria. RMC: CBCT ~6–25% depending on population and criteria; PR detects substantially fewer. AL: presence and reported prevalence are threshold-dependent; length commonly 1–3 mm on CBCT (plane/definition influence estimates); margins should be individualized from patient-specific CBCT. AMF: often missed on PR; adult CBCT cohorts commonly ≈10–12% under rigorous definitions. HMC: definition-dependent; MC–crest ≤4–5 mm at the target site indicates limited vertical clearance and higher risk. MRI (3T): selective soft-tissue tract visualization; pooled “prevalence” is not appropriate.

### 3.1. Study Selection and Characteristics (PRISMA-Lite)

A qualitative synthesis included imaging and anatomic studies (PR/CBCT/MRI) published between 2000 and 2025. The primary sources of heterogeneity were CBCT voxel size (≈0.12–0.40 mm), operational definitions of variants (e.g., confirmation in ≥2 planes for bifid canals), thresholds for the AL (see Box 1). No meta-analysis was performed due to inconsistency in definitions and acquisition parameters (see Table 1 for study-level detail).

### 3.2. Imaging Parameters Overview

**Figure 2:**
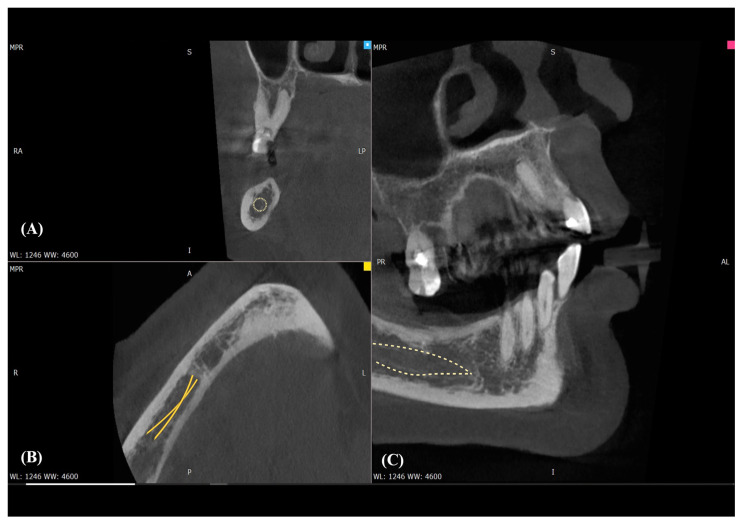
CBCT multiplanar visualization of the mandibular canal (MC). (**A**) Axial view showing the corticated cross-section of the mandibular canal (yellow dotted circle). (**B**) Sagittal reconstruction demonstrating two parallel corticated tracts suggestive of a bifid configuration of the mandibular canal (yellow lines). (**C**) Coronal reconstruction outlining the course of the main mandibular canal within the mandibular body (yellow dotted outline).

PR served as an initial screening tool. CBCT substantially increased detectability of bony variants (e.g., BMC, RMC, AMF) and enabled reproducible morphometrics (e.g., AL length). MRI at 3 T (including DTI where applicable) was used selectively to delineate the inferior alveolar neurovascular bundle in soft tissue when clinical questions extended beyond CBCT’s capability (see Table 1). Figure 2 illustrates how CBCT MPR delineates the canal course and allows direct measurement of crest–MC clearance, which PR cannot provide.

### 3.3. Bifid/Trifid Mandibular Canal (BMC/TMC)

**Figure 3:**
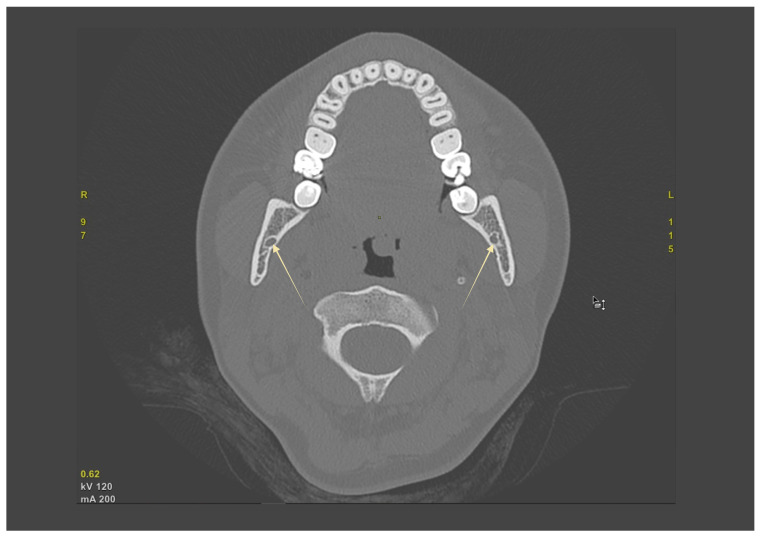
Axial CBCT demonstrating the mandibular canal (MC) bilaterally. Corticated cross-sections of the mandibular canals (yellow arrows) are visible within the mandibular bodies on both sides, allowing direct assessment of canal morphology and symmetry.

Across CBCT cohorts, BMC prevalence typically ranged from single-digit to low double-digit percentages, with extremes explained by definition and voxel size. Trifid canals were uncommon (≈1–2%). PR consistently underestimated canal branching compared with CBCT. Clinical implications for local anesthesia, osteotomy planning, and hemostasis are summarized in Table 2; study-level prevalence and definitions appear in Table 1. In practice, axial CBCT is useful for screening bifid or retromolar branches and for bilateral comparison (Figure 3).

### 3.4. Retromolar Canal (RMC)

RMC was identified on CBCT from low to mid-double-digit rates depending on population and criteria, with representative cohorts reporting frequencies around the mid-teens to mid-twenties. The presence of an accessory neurovascular bundle in the retromolar triangle increases the risk of bleeding and sensory disturbance; planning and intraoperative modifications are outlined in Table 2. Individual study frequencies and morphometrics are provided in Table 1.

### 3.5. Anterior Loop (AL) of the Mental Nerve

In implantology, the AL of the mental nerve is often analyzed as a standalone topic; we retain it here to enable cross-variant comparison within a single, clinically oriented workflow. AL detection and measured lengths varied with the applied threshold (commonly ≥1–3 mm) and the measurement plane. In clinical practice, the safety margin in the premolar (3–5) region should be individualized from CBCT rather than assumed universally. Threshold-specific AL data are listed in Table 1, and recommended surgical adjustments are summarized in Table 2.

### 3.6. High-Positioned Mandibular Canal (HMC)

A reduced vertical distance from the MC to the alveolar crest was reported with definition-dependent frequency and was more likely in resorbed, edentulous mandibles in several series. Clinically, HMC narrows the osteotomy corridor and may necessitate shorter implants, altered trajectories, or navigation support. See Table 1 for study definitions/metrics and Table 2 for procedural adaptations.

### 3.7. Accessory Mental Foramen (AMF) and Accessory Canals

AMF was more commonly detected on CBCT than on PR. Because AMF represents additional exits of the mental neurovascular bundle, lateral cortical drilling and corticotomy trajectories should be planned to avoid these paths. Prevalence and imaging criteria are compiled in Table 1; risk and technique modifications are presented in Table 2.

### 3.8. Synthesis: Variant-Aware Imaging Workflow (PR → CBCT → Selective MRI)

We do not advocate routine CBCT for all posterior mandibular procedures; instead, CBCT is obtained when predefined criteria are met and when results are reasonably expected to change management, consistent with ALARA/ALADA. CBCT triggers (examples): equivocal or suspicious PR; planned implant near the mental foramen with uncertain AL; reduced MC–crest distance at the target site (e.g., ≤4–5 mm); PR/clinical suspicion of BMC/RMC/AMF; complex third-molar relation to MC; repeated IAN block failures; unexplained or persistent neurosensory symptoms.

- PR for routine screening in straightforward cases without red flags.
- CBCT when planning implants in the premolar region (3–5), when the canal is close to the planned osteotomy/implant, when BMC/RMC/AMF is suspected, after repeated IAN block failures, or for complex mandibular third-molar surgery (see Table 2 for how variants alter practice).
- MRI (selective) for soft-tissue questions about the inferior alveolar bundle or when CBCT does not explain persistent neurosensory symptoms.

All imaging decisions were aligned with ALARA/ALADA principles. Study-specific thresholds, parameters, and outcomes are consolidated in Table 1; associated clinical adjustments appear in Table 2.

### 3.9. Clinical Box: Anesthesia & MC Variants—Box 2

To translate variant-aware findings into practice, the following clinical box highlights anesthesia-related red flags and procedure-modifying implications; actionable pairings are summarized in Table 2.

Box 2 **Anesthesia & MC Variants.**

**Checkpoint**

**Cue/Red Flag**

**Action (Concise)**

**See**
IANB failuresRepeated/atypical responseMap variants on CBCT
Table 2
Suspected BMC/TMC/AMFPR hint or clinical patternTargeted CBCT; adjust plan
Table 2
Premolar region (3–5)Margin depends on ALMeasure AL; individualize margin
Table 2
High-positioned MC (HMC)Short MC–crest distanceShorter implant; altered angle
Table 2
Third-molar/posterior surgeryPossible RMCModify flap; prepare hemostasis
Table 2
Discordant findingsAtypical paresthesiaCBCT to map variant anatomy
Table 2
Anesthesia adjunctsPersistent difficultyConsider intraosseous/intraligamentary
Table 2
Documentation/consentVariant identifiedRecord baseline & postop sensibility
Table 2

### 3.10. Imaging Workflow

**Figure 4:**
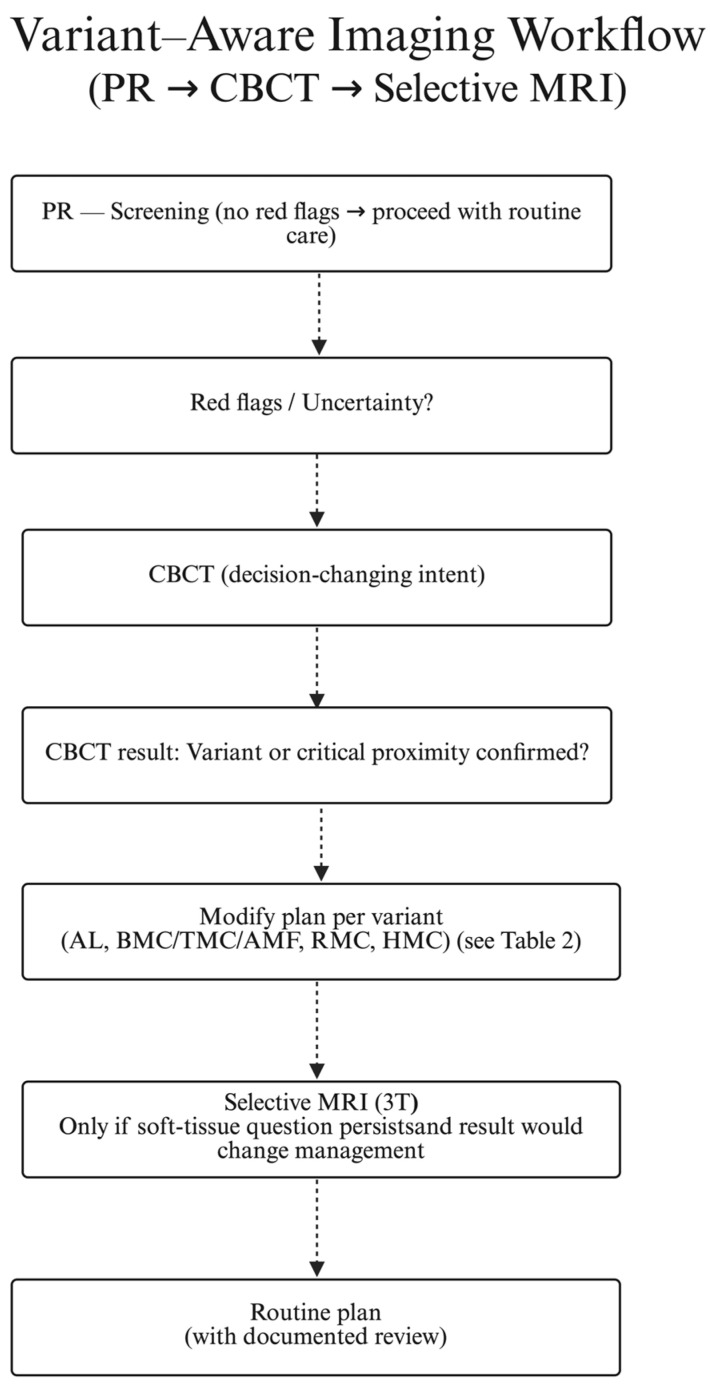
Variant-aware imaging workflow (PR → CBCT → selective MRI). Sequential decision pathway illustrating when to proceed from panoramic radiography to CBCT or MRI based on red flags and clinical relevance, following ALARA/ALADA principles.

Building on the above, the variant-aware imaging workflow specifies when PR suffices, when CBCT is decision-changing, and when selective MRI adds value, in line with ALARA/ALADA (see Figure 4 and Table 2). Accordingly, CBCT use is indication-driven rather than universal, aligned with ALARA/ALADA stewardship and consistent with recent imaging-led anatomical frameworks [30,31,32].

### 3.11. Landfald Clinical Framework

We next outline the LCF, a hypothesis-generating tool linking common variants to imaging steps and procedural adjustments, with pragmatic thresholds and illustrative vignettes; it is not proposed as a new standard (see Table 2). Thresholds and confirmation rules are summarized in Box 1.

Illustrative clinical vignettes showing LCF-guided changes in implant planning (AL) and third-molar surgery (RMC) are provided in Section 7.4.

Validation statement: The LCF requires formal validation—dual-reader reliability (kappa/ICC), construct/criterion validity against surgical findings, and external replication, before any consideration as a standard. Figure 4 depicts the overall imaging workflow; variant–technique pairings are consolidated in Table 2. Operational thresholds (Box 1) are applied at the target site—with confirmation in ≥2 orthogonal planes—and guide decision-changing imaging and patient-specific margins within the LCF.

A proposed roadmap for validating the framework—covering inter-reader reliability, construct validity, and external replication- is outlined in Box 3.

Box 3 **alidation plan for the LCF.**

**(i) Inter-reader reliability.** Two independent readers, blinded to each other and outcomes; calibration on a small training set (not analyzed), then repeated reads of a random subset after a wash-out. Report κ (categorical calls:

BMC/RMC/AMF/HMC/AL presence) and ICC(2,1) (linear measures: AL length; MC–crest clearance), each with 95% CIs and number of cases/segments.

**(ii) Construct/criterion validity.** Test convergence with intra-operative findings and patient outcomes. Predefine decision-change endpoints (e.g., implant size/trajectory change; altered flap/osteotomy; avoidance of nerve injury). Evaluate performance of operational thresholds (AL ≥ 1–3 mm; HMC ≤ 4–5 mm, confirmed in ≥2 planes) against surgical margins/neuropraxia outcomes.

**(iii) External replication.** Prospective multi-center registry using standard data fields (modality, voxel size, plane confirmation, variant codes, thresholds, decision changes, complications). Report adherence to ALARA/ALADA triggers and patient-level outcomes.

## 4. Embryological and Developmental Basis of Variants

Anatomical variants of the MC and IAN originate from embryological transformations involving Meckel’s cartilage, which is derived from the first pharyngeal arch. Meckel’s cartilage becomes apparent around the fifth embryonic week (Carnegie stage 13; approximately day 32), acting as a cartilaginous scaffold for mandibular development. Ossification of the mandible begins adjacent to this cartilage around gestational week 10 [33,34].

During fetal development, Meckel’s cartilage undergoes partial regression, with only residual structures persisting postnatally as the malleus and sphenomandibular ligament. The embryonic IAN develops from multiple nerve bundles originating from neural crest cells. Incomplete fusion of these bundles during ossification may result in BMC or TMC [33,35,36]. Likewise, the RMC likely represents a persistent pathway of secondary nerve branches supplying developing dentition or masticatory muscle insertions.

Vascular development also plays a critical role in variant formation. Embryonic vascular channels penetrating the mandibular bone can persist as accessory foramina, such as accessory mental or lingual foramina. A notable example is the “conduct of Serres”, a temporary fetal canal that transmits the embryonic vein of Serres; in some cases, this structure persists postnatally as an accessory MC [37,38].

This developmental perspective allows logical categorization of variants into

(1) nerve-splitting variants (e.g., bifid and trifid canals);
(2) vascular-based variants (e.g., accessory foramina);
(3) positional growth variants (e.g., high-positioned MC).

These should be considered normal developmental outcomes rather than pathological anomalies. These developmental mechanisms are summarized in Table 3, which categorizes major anatomical variants of the MC and IAN according to their embryological origins and associated clinical implications.

## 5. Imaging Modalities and Detection of Variants

### 5.1. PR Versus CBCT

**Figure 5:**
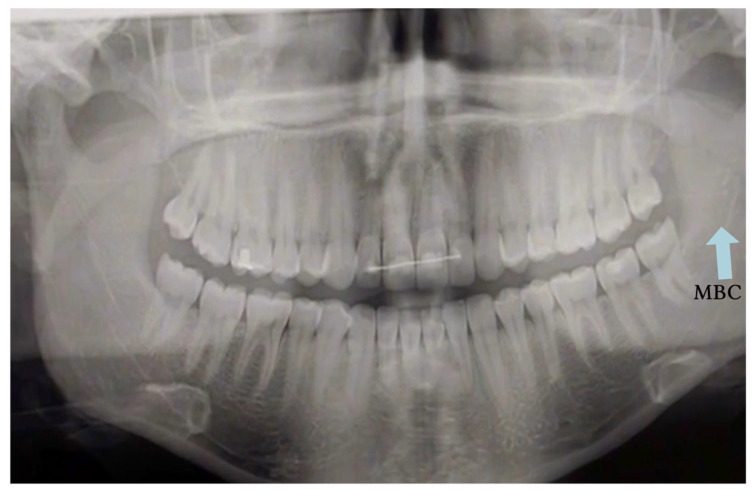
Panoramic radiograph (PR) showing a suspected bifid mandibular canal (BMC; blue arrow). The blue arrow indicates the cortical outline suggestive of canal duplication. PR alone cannot confirm this finding; CBCT is recommended when confirmation would change management (CBCT not shown).

Traditional PR has significant limitations in accurately detecting MC anatomical variations. Earlier studies relying exclusively on panoramic imaging reported very low prevalences typically below 1% for bifid MC, largely due to anatomical superimposition and the inherent distortion of two-dimensional imaging [4,5]. Figure 5 illustrates why PR can be equivocal for canal variants; in such scenarios, CBCT confirmation is recommended if it would alter the treatment plan.

The advent of CBCT has markedly improved detection sensitivity. Its three-dimensional capability eliminates structural overlap, enabling accurate assessment of BMC, TMC, AL, RMC, and accessory foramina, which are often missed on PR [8,12]. For detailed prevalence data and diagnostic implications see Table 1 and Table 2.

Given these considerations, CBCT should be obtained selectively—when predefined, decision-changing criteria are met (see Figure 4): suspected canal variants (BMC/TMC/RMC/AMF) on PR or clinical grounds; implant planning in the premolar region (3–5) where AL length influences safety margins; a short MC–crest distance at the planned osteotomy/implant site; repeated IANB failure; or complex mandibular third-molar surgery. This approach aligns with ALARA/ALADA and current evidence [25,39].

### 5.2. Magnetic Resonance Imaging (MRI) and Advanced Techniques

MRI, especially at higher field strengths such as 3T, complements CBCT by visualizing the neurovascular bundle (soft-tissue tract) within the MC region. Unlike CBCT, MRI can detect subtle soft-tissue structures, including branches of the IAN, even without corresponding distinct bony canals. Krasny [11] utilized 3T MRI to identify multiple nerve branches within apparently single canals, offering explanations for certain unexplained anesthetic failures or neuropathies encountered clinically.

Recent MRI studies further illustrate this selectivity. Öçbe and Borahan [7] demonstrated accessory-branch soft-tissue tract visualization in question-driven MRI examinations; because MRI does not depict corticated bony canals in the CBCT sense and these cohorts are selectively indicated, population-level “prevalence” figures are not appropriate and are not directly comparable to CBCT estimates. Furthermore, emerging diffusion tensor imaging tractography methods have shown significant promise for detailed nerve mapping, potentially enhancing preoperative planning accuracy by visualizing precise nerve trajectories [20]. Nevertheless, rigorous anatomical validation through cadaveric studies remains necessary before widespread clinical adoption.

However, MRI’s clinical utility is currently limited by higher costs, longer scanning times, limited availability, and sensitivity to metallic artifacts, restricting its routine dental use. Thus, MRI is recommended selectively, primarily when complex nerve anatomy is suspected or when CBCT findings are inconclusive.

### 5.3. Capabilities and Limitations of Imaging Modalities

Each imaging technique presents unique strengths and limitations in evaluating MC variants. CBCT excels in demonstrating detailed bony anatomy with excellent spatial resolution and minimal distortion, reliably depicting accessory canals larger than approximately 0.1–0.2 mm in diameter. However, CBCT may miss smaller neural branches lacking distinct bony delineation. In contrast, MRI excels in soft-tissue differentiation, clearly depicting nerves and small vascular structures even in the absence of a distinct bony canal. However, MRI is disadvantaged by lower spatial resolution for bone, limited accessibility, higher costs, and susceptibility to artifacts.

Given these considerations, a recommended clinical imaging protocol involves:

**Step 1: PR screening**—routine, straightforward cases without red flags.

**Step 2: Selective CBCT**—obtain only when predefined, decision-changing criteria are met (suspected BMC/TMC/RMC/AMF; premolar region 3–5 where AL affects margins; short MC–crest distance at the planned site; repeated IANB failure; complex M3) (see Figure 4).

**Step 3: Selective MRI (3T)**—reserved for unresolved soft-tissue questions when the result would change management.

### 5.4. Clinical Implications of Radiological Findings

The accurate identification of MC and IAN variants using advanced imaging modalities has profound clinical and medicolegal implications. Conventional PR, despite its historical utility in screening, consistently underestimates anatomical variability due to low spatial resolution and structural superimposition, with BMC detection rates rarely exceeding 1% [4,5].

By contrast, CBCT markedly enhances diagnostic sensitivity. In adult cohorts, typical BMC frequencies on CBCT fall in the single-digit to low double-digit range; higher values reported in some series reflect finer voxel sizes and stricter operational definitions, whereas PR detects substantially fewer. Trifid canals remain uncommon (≈1–2%) [3,8,22,25].

MRI, especially at 3 Tesla, complements CBCT by enabling direct visualization of soft-tissue structures, including IAN branches and neurovascular pathways, even in the absence of visible bony canals. Studies using high-resolution MRI have identified accessory neural branches within apparently single canals, providing explanations for anesthesia failure and unexpected neuropathy [7,11]. Moreover, advanced techniques such as DTI offer promising yet still experimental nerve-mapping capabilities, warranting further anatomical validation before clinical adoption [20].

Clinically, preoperative identification of variants such as BMC, AL, accessory foramina, or HMC allows for tailored surgical strategies, thereby reducing the risks of nerve trauma, hemorrhage, and postoperative complications. These findings inform decisions regarding surgical access, implant angulation, anesthetic technique, and flap design.

Furthermore, thorough radiological evaluation and documentation play a crucial role in medicolegal risk mitigation. From a medicolegal perspective, advanced imaging—particularly CBCT and, in selected indications, MRI—should be obtained when it is reasonably expected to change management in high-risk mandibular regions, consistent with ALARA/ALADA principles.

In summary, CBCT should be the primary modality for variant detection and surgical planning, while MRI serves as a valuable adjunct in complex cases. Their combined use supports safer, variant-aware oral and maxillofacial surgery, improving patient outcomes and medico-legal defensibility. The distinct diagnostic capacities of various imaging modalities used in assessing MC variants are summarized in Table 4, which contrasts their sensitivity, tissue visualization capabilities, radiation profiles, and clinical limitations. This structured overview facilitates optimal modality selection depending on the anatomical and surgical context.

## 6. Surgical and Clinical Implications

This section translates variant-aware imaging into domain-specific procedural adaptations for local anesthesia, implantology, mandibular third-molar surgery, and osteotomies. Consolidated, procedure-level guidance—together with decision-change imaging triggers (PR → selective CBCT; MRI reserved for soft-tissue pathway questions)—is summarized in Table 5 (Domain-specific adaptations by variant). For a rapid, variant-first view of risk → mechanism → imaging trigger → planning, see Table 2 (Quick Clinical Keys); per-study thresholds and parameters remain in Table 1.

### 6.1. Local Anesthesia and Nerve Blocks

Undiagnosed MC variations are a common cause of failed IAN blocks. Bifid MCs or accessory branches near the mandibular foramen may result in incomplete anesthesia, as conventional IAN blocks may only affect the main nerve trunk, leaving accessory branches intact [10]. Advanced imaging, particularly CBCT, improves detection of such variants [3,21], while MRI may reveal subtle branching patterns not visible on conventional imaging [7,11]. This justifies its selective use in patients with recurrent IANB failures. In such cases, clinicians should consider alternative anesthetic approaches such as Gow-Gates, Akinosi-Vazirani, or targeted infiltration at accessory foramina sites [19,40]. See Table 5 for variant-specific anesthesia adaptations and decision-change triggers.

### 6.2. Third-Molar Surgery

**Figure 6:**
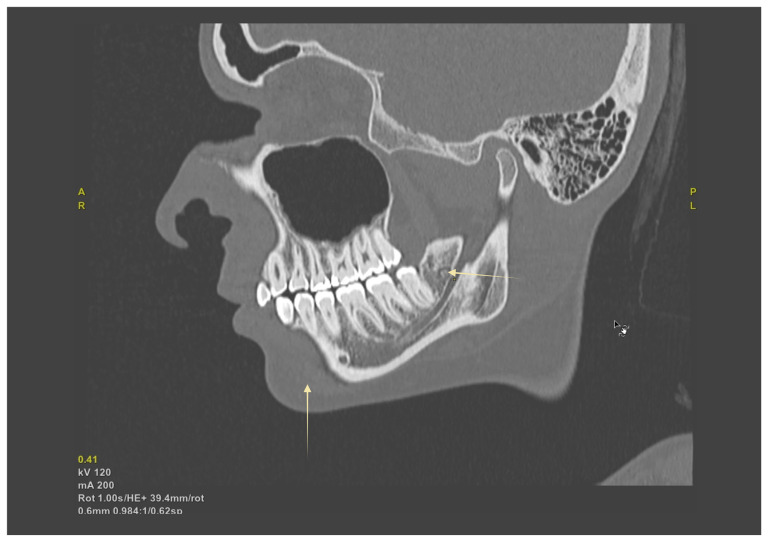
Oblique-sagittal CBCT reconstruction demonstrating the course of the mandibular canal (MC). The yellow arrows indicate the canal trajectory within the mandibular body and its proximity to the molar roots, serving as key landmarks for preoperative assessment and surgical planning.

Surgical extraction of mandibular third molars poses a significant risk for IAN injury, especially in the presence of RMCs or HMC. Undiagnosed RMC variants can cause unexpected intraoperative hemorrhage and postoperative neuropathy if transected during flap elevation or bone removal [12,16]. Similarly, an HMC located close to tooth apices limits surgical space, increasing the risk of nerve stretch or compression injuries [2,28]. Preoperative CBCT imaging significantly improves the detection of these variations [8,12,25], facilitating tailored surgical techniques, such as coronectomy to avoid nerve entrapment or modified flap designs to circumvent accessory nerve branches [3,39]. When proximity is high, prioritize conservative ostectomy, consider coronectomy, and plan flap/suture strategy to avoid accessory foramina and the retromolar foramen (see Table 5). Figure 6 shows a sagittal CBCT with an impacted third molar and the MC in close proximity, illustrating why CBCT mapping can alter the choice of approach (e.g., coronectomy, modified flap).

### 6.3. Dental Implantology

Accurate identification of MC and IAN variations is critical during posterior mandibular implant planning. Variants such as an HMC restrict vertical bone availability, increasing the risk of nerve injury during osteotomies [26]. AL of the mental nerve extending ≥3 mm anterior to the MeF require larger safety margins to avoid nerve impingement or injury [18,24]. Reported AL prevalence and measured length vary with imaging modality, voxel size, measurement plane and operational definition; consequently, implant safety margins in the premolar (3–5) region should be individualized from patient-specific CBCT measurements rather than set as a fixed universal rule. When the loop cannot be delineated with confidence, a more conservative posteriorization or a shorter fixture—and, where appropriate, guided surgery—are prudent. Maintain a conservative vertical and horizontal safety margin to the canal and mental foramen/loop; adjust implant length, trajectory, or site accordingly. Any uncertainty about loop length or course should trigger a posterior shift or shorter fixture (see Table 5). Additionally, accessory mental foramina may contain small nerve branches leading to localized numbness or hemorrhage if intersected during implant osteotomies [11,19]. Buccolingual bifid canal variants can complicate lateral drilling or ridge modifications due to unpredictable nerve positions [41,42]. Thus, preoperative CBCT imaging is essential for precise planning of implant length, angulation, and position [1,8].

### 6.4. Oncologic and Reconstructive Surgery

In mandibular resections and reconstructive procedures, precise knowledge of MC anatomy is essential. Unrecognized BMC or TMC variants increase the risk of unintended nerve transection or excessive bleeding during segmental osteotomies [3,22]. Similarly, accessory foramina and RMC may serve as conduits for postoperative bleeding or infection [37,38]. Preoperative CBCT is vital for mapping these anatomical variants, while MRI may offer additional soft-tissue insights in complex cases [7,11]. Accurate imaging allows for surgical plan adjustments that preserve nerve integrity and minimize vascular injury. In orthognathic surgeries such as sagittal split ramus osteotomies, medial or lingual deviation of the MC substantially increases the risk of nerve injury, necessitating careful modification of osteotomy techniques [27,28].

### 6.5. Postoperative Complications and Medicolegal Considerations

Undiagnosed MC and IAN variants frequently contribute to postoperative complications such as prolonged paresthesia, dysesthesia, and neuroma formation. Partial injury to bifid or accessory nerve branches during extractions or osteotomies may result in persistent neuropathy or chronic pain [22,39]. Similarly, injury to accessory foramina can lead to localized numbness or vascular complications. From a medicolegal perspective, advanced imaging, particularly CBCT and, in selected indications, MRI—should be obtained when it is reasonably expected to change management in high-risk mandibular regions, consistent with ALARA/ALADA principles [1]. Failure to identify anatomical variants may expose clinicians to liability, especially when preventable complications occur. Structured, variant-aware documentation templates can support consistent preoperative risk assessment and postoperative follow-up. Postoperative follow-up should include active screening for atypical neurosensory symptoms, with targeted CBCT or MRI when variant anatomy is suspected. Early detection can guide management strategies, including focused nerve stimulation and neuromodulation [7,20]. The clinical consequences of anatomical variants of the MC and IAN vary according to the surgical domain and variant type. These implications are summarized in Table 5, which outlines the variant-specific risks, recommended imaging modalities, and procedural precautions relevant to contemporary oral and maxillofacial practice. For medicolegal clarity, clinicians should document the indication and decision-change trigger for imaging (e.g., equivocal PR, proximity below threshold, suspected AL).

## 7. LCF Clinical Framework and Workflow

### 7.1. Rationale and Scope

**Figure 7:**
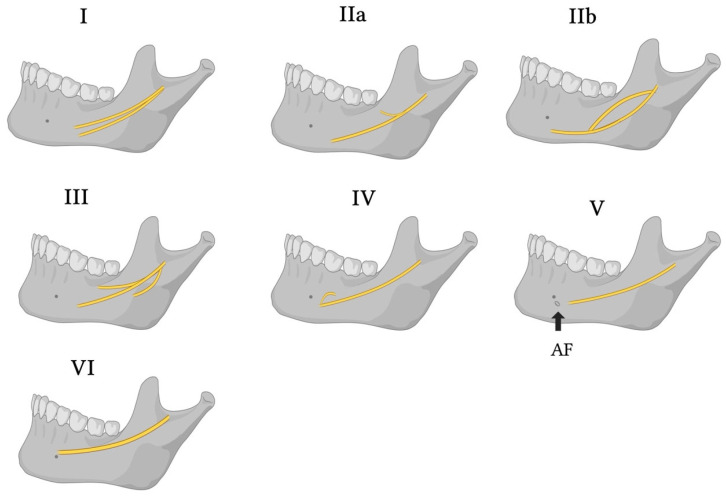
LCF-guided imaging variants. Panels (**I**–**VI**) illustrate representative mandibular canal configurations used within the Landfald Clinical Framework to guide imaging and planning (e.g., single course; bifid/trifid or retromolar branching; anterior loop–dominant course; high-positioned canal; accessory exits). The yellow line marks the inferred inferior alveolar neurovascular pathway/mandibular canal; AF denotes an accessory foramen.

Earlier descriptive systems—particularly those relying on PR—offered limited practical guidance for risk assessment and planning: BMC were often underreported (<1%) due to superimposition and 2D distortion [20,35]. With cross-sectional imaging, visualization improved substantially, revealing higher rates of BMC/TMC, RMC/RMF and accessory foramina, and more intricate IAN branching [2,6,18,26,40]. Against this background, we present the LCF clinical framework: a hypothesis-generating, variant-aware tool that links imaging features to decision-relevant thresholds and procedure-specific precautions. It is not a de novo anatomical taxonomy or standard. Point-of-use thresholds and confirmation criteria are summarized in Box 1; procedure-specific adaptations are summarized in Table 5. These thresholds anchor the decision-changing triggers summarized in Section 7.2. Representative variant patterns are illustrated in Figure 7.

### 7.2. Clinical Justification

LCF prioritizes situations in which imaging changes management. Based on the operational thresholds in Section 7.1, imaging is escalated only when the following decision triggers are present:

- PR conflicting/equivocal findings.
- Planned implant in variant-rich zones (premolars 3–5; retromolar/ramus).
- Uncertain MeF location on PR.
- AL ≥1–3 mm on CBCT (confirmed in ≥2 planes).
- Planned ramus/sagittal split or complex M3 procedures.
- MC–crest clearance ≤4–5 mm at the planned site (CBCT, ≥2 planes).
- Repeated IANB failure.

(Selective MRI is reserved for soft-tissue tract visualization when the result would change management.)

In practice, this means (1) PR for screening; (2) CBCT selectively when predefined, decision-changing criteria are met; and (3) selective MRI where soft-tissue tract visualization could alter the plan. LCF maps common variants to concrete adjustments—e.g., adapt osteotomy corridors/drill trajectories for bifid/trifid canals [12,16]; individualize margins in the premolar (3–5) region where AL length (commonly ≥1–3 mm) affects safety [18,24]; reconsider implant length/angulation or use guidance when the MC–crest distance ≤4–5 mm at the target site; and exercise caution around RMC/AMF because of bleeding/neurosensory risk [15,19,29]. See Figure 4 (workflow) and Table 2.

### 7.3. Variant-Aware Clinical Workflow

Step 1—PR (screening). Look for overt anomalies (e.g., radiolucent duplication/retromolar tract suggestion) [4].

Step 2—CBCT (selective). Obtain only when decision-changing criteria are present: suspected BMC/TMC/RMC/AMF on PR or clinical grounds; short MC–crest distance at the planned site; AL influencing premolar planning; repeated IANB failure; complex M3—consistent with ALARA/ALADA principles [25,39].

Step 3—MRI (selective). Selective MRI (3T): soft-tissue tract visualization of neurovascular bundles; consider it only when equivocal CBCT or a soft-tissue question would change the plan [7,11,20].

Step 4—LCF notation in the record. Use concise patient-level labels (e.g., “LCF-BMC buccolingual; LCF-RMC unilateral; LCF-HMC at 36”).

Step 5—Procedure tailoring (examples).

- LCF-BMC/LC-TMC: adjust osteotomy corridors and drilling trajectories; consider multi-site anesthesia [3,12].
- LCF-RMC: modify flap design; anticipate/control retromolar vessels; consider piezosurgery [12,16,23].
- LCF-AL (≥1–3 mm): individualize margins in premolars (3–5) from CBCT; avoid fixed distances a priori [18,24].
- LCF-AMF: avoid osteotomies across corticated accessory foramina; consider guidance [19,29].
- LCF-HMC (MC–crest ≤4–5 mm at target site): reconsider implant length/angulation or use guided surgery; avoid vertical crest-adjacent cuts [2,26].

Step 6—Post-event documentation. If unexpected neurosensory change occurs, document LCF category, preoperative imaging and intraoperative modifications; consider targeted postoperative imaging when clinically justified.

### 7.4. Clinical Vignettes (LCF in Practice)

Vignette 1—Implant in the 3–5 region (suspected AL). PR is equivocal for an AL. CBCT confirms AL = 2.1 mm, measured and confirmed in ≥2 orthogonal planes (within the ≥1–3 mm operational threshold). Before LCF: a 4.3 × 10 implant with a standard safety margin was planned. LCF-guided change: switch to 3.8 × 8, adjust trajectory to preserve a patient-specific offset beyond the AL, and use guided surgery; MRI is not indicated (no soft-tissue question).

Vignette 2—Third molar with suspected RMC. PR is unclear for accessory branching. CBCT maps an RMC coursing toward the retromolar fossa. Before LCF: routine triangular flap and rotary osteotomy were planned. LCF-guided change: modify the flap and elevation, apply pre-emptive hemostasis, use a conservative osteotomy corridor (consider piezosurgery), and avoid suction trauma; MRI is not indicated (no soft-tissue question).

Vignette 3—Recurrent IAN block failure (suspected BMC). PR is non-diagnostic for variant branching. Selective CBCT (isotropic voxel ≈ 0.20–0.25 mm) demonstrates a BMC with a buccal accessory limb toward the molar apex, confirmed in ≥2 orthogonal planes. Before LCF: repeat conventional IAN block and routine flap/osteotomy were planned. LCF-guided change: switch to Gow–Gates/Vazirani–Akinosi plus targeted buccal/lingual infiltrations along the accessory tract; modify flap/osteotomy corridor to avoid the canal and prepare hemostasis; MRI not indicated (no soft-tissue question).

### 7.5. Clinical Impact

Embedding LCF encourages anticipatory rather than reactive decisions and improves cross-team communication. By stating when and why imaging alters the plan—and by using explicit thresholds (e.g., AL ≥ 1–3 mm, MC–crest ≤ 4–5 mm)—LCF aims to increase predictability and patient safety without promoting indiscriminate imaging (Vranckx, 2022) [39].

## 8. Future Directions and Innovations

Critical appraisal: Selective MRI may assist when CBCT findings are equivocal or detailed neurovascular mapping is required, but several constraints limit immediate, broad adoption: (1) cost and access—availability, scheduling and reimbursement vary across settings; (2) validation gaps—there are few prospective, multi-center studies with inter-rater reliability and clinically meaningful endpoints (e.g., neurosensory outcomes); (3) heterogeneity—CBCT parameters (voxel size, FOV, kVp/mA) and MRI protocols (field strength, sequences) are not standardized, which impairs comparability; (4) radiation stewardship—CBCT should follow ALARA/ALADA with decision-changing thresholds. Addressing these points is prerequisite to guideline-level recommendations.

Despite recent progress in understanding MC and IAN variants, several challenges remain in imaging standardization, clinical implementation, and education. Addressing these gaps may enhance procedural safety and long-term outcomes.

### 8.1. International Variant Registries

There is a critical need for large-scale international registries of CBCT and MRI scans annotated for MC variants. Current studies are often limited by regional sampling. Multi-center repositories would allow for robust mapping of global prevalence and support research into demographics and clinical risk stratification [8,22].

### 8.2. MRI Tractography and Validation

Diffusion tensor imaging tractography enables non-invasive mapping of IAN trajectories, revealing complex branching patterns not visible on CBCT. Cadaveric validation is essential to ensure clinical reliability [20].

### 8.3. Augmented Reality and Intraoperative Navigation

Augmented reality (AR) may provide real-time visualization of MC variants during surgery by integrating CBCT data intraoperatively. Preliminary studies suggest improved surgical accuracy, particularly in variant-rich zones. He et al. [43] demonstrated a 57–62% reduction in osteotomy placement error using AR guidance in mandibular distraction procedures. Similarly, a systematic review by Puleio et al. [44] concluded that AR consistently enhances precision across dental surgical interventions.

### 8.4. Educational Integration

Anatomical variability should be reflected in curricula and surgical training. LCF-based frameworks and 3D-printed CBCT-derived models offer enhanced simulation opportunities. A case-based, risk-adapted approach can better prepare trainees [22].

A structured roadmap for innovation in MC and IAN management is summarized in Table 6. This table outlines the key research and clinical directions, including AI-based variant detection, MRI validation studies, and surgical navigation tools.

## 9. Conclusions

This review underscores that anatomical variants of the mandibular canal (MC) and IAN) are far more prevalent than traditionally recognized and carry significant clinical implications. With the advent of advanced imaging modalities, especially CBCT and high-resolution MRI, variants such as bifid and trifid canals, anterior loops, and accessory foramina are now acknowledged as common features, not anomalies.

To translate this anatomical variability into safer clinical practice, we propose the LCF—a system directly linking variant types with procedural risks and recommended imaging protocols. This variant-aware workflow improves preoperative planning, reduces iatrogenic complications, and enhances patient outcomes across surgical, implantological, and anesthetic interventions.

An imaging-led pathway (PR → CBCT → selective MRI) aligned with the LCF translates canal variants into concrete risk–adaptation pairs across anesthesia, implantology, third-molar surgery, and osteotomies. Next steps are prospective, multi-center validation, protocol harmonization, and identifying clear decision-change triggers under ALARA/ALADA.

In sum, integrating LCF and advanced imaging into routine care represents a paradigm shift in oral and maxillofacial surgery—from reactive intervention to proactive, evidence-guided practice.

## Figures and Tables

**Table 1:** Per-study characteristics and outcomes (complete rows).

Study (First Author, Year)	Modality	Voxel/Sequence	Operational Definition/Threshold	Population	N (Unit)	Variant	Prevalence (%)	Morphometrics (Units)	Reader Method
Langlais [4]	PR	—	Duplicate/parallel corticated tract visible on panoramic radiograph	Adults; routine dental radiography	6000 PRs	BMC	1.0 (57/6000)	—	NR
Kuribayashi [14]	CBCT	0.125 mm (3DX micro CT); 1 mm slice	BMC present; Nortjé-type classification; ≥2-plane confirmation	Adults (mean age 33; 18–74); impacted third molars	252 pts/301 sides	BMC	15.6 (47/301)	Mean canal diameter 1.68 mm (0.88–3.40); main canal 3.28 mm (2.02–4.63)	Two oral radiologists; independent readings; consensus after discussion
Sisman [15]	PR vs. CBCT	PR: —; CBCT: 0.15 mm voxel (NewTom 5G typical)	RMC present (comparison PR vs. CBCT)	Adults (632 individuals; 947 hemimandibles) with impacted M3 (mean age 27.47 ± 8.74 years)	632 pts/947 hemimandibles	RMC	CBCT: 26.7; PR: 3.06	Canal height 11.4 ± 2.61 mm; horizontal distance to second molar 15.45 ± 3.12 mm; origin width 2.24 ± 0.94 mm; exit width 1.64 ± 0.64 mm	Two dentomaxillofacial radiologists; independent evaluation; consensus when disagreement; measurements repeated after 2 weeks
Shen [16]	CBCT	0.155 mm (AZ3000; 85 kVp, 12.5 mA; FOV 70 mm)	RMF present (distinct foramen/tract in ≥2 planes)	Adults; Taiwan, medical center	68 hemimandibles	RMC/RMF	10.3 (7/68)	RMF Ø 1.41 ± 0.30 mm; MeF–RMF 11.57 ± 2.70 mm; RMF–crest 13.62 ± 1.34 mm	NR
von Arx [12]	CBCT	NR	RMF present on CBCT (corticated tract/foramen)	Adults; imaging study (CH)	100 pts/121 sides	RMC/RMF	25.6 (sides)	Distance to second molar = 15.16 ± 2.39 mm; canal height = 11.34 ± 2.36 mm; canal width = 0.99 ± 0.31 mm	NR
do Nascimento [17]	CBCT	0.25 mm (i-CAT)	AL present; length measured in orthogonal planes	Adults; Brazil (radiology clinic)	250 pts/500 hemimandibles	AL	41.6	AL length mean 1.1 ± 0.8 mm (0.25–4.00)	Two trained and calibrated observers; consensus after discussion
Uchida [18]	Anatomy vs. CBCT (subset)	—	AL length measured directly (cadaveric)	38 cadavers/75 hemimandibles	75 hemimandibles	AL	—	AL mean 1.5 ± 1.4 mm (0–6)	Direct measurements
Mostafavi [19]	CBCT	NR	AMF per CBCT criteria; foramen-level reporting	Adults; single center	2082 CBCTs	AMF	11.8	NR (size not reported)	NR
Krasny [11]	MRI (3T)	3T; T1/T2/TIRM (conventional)	Visualization of neurovascular tract (soft-tissue)—no bony canal prevalence	Adults; dentate	64 patients	Soft-tissue tract	—	NR (soft-tissue only)	NR
Kotaki [20]	MRI-DTI (3T)	3T MRI; single-shot EPI DTI with STIR fat suppression; 3D T1 MP-RAGE; 16-channel head/neck coil	Neurovascular bundle course mapped by fiber tracking	Healthy volunteers	46 volunteers (92 IANs)	Soft-tissue tract	—	NR (soft-tissue only)	NR

Notes: NR = not reported; em dash (—) denotes not applicable (e.g., MRI rows reflect soft-tissue tract visualization rather than CBCT-style bony canal prevalence). Percentages rounded to one decimal place where applicable. Voxel size refers to CBCT isotropic voxel.

**Table 2:** Quick Clinical Keys for variant-aware decision-making.

Variant	Key Clinical Risk	Mechanism	Imaging Trigger	Planning/Actions	Key Sources
Bifid/Trifid mandibular canal (BMC/TMC)	Failed anesthesia; neurosensory injury; intraoperative bleeding	Duplicated neurovascular pathway with accessory limbs/exits	PR equivocal or repeated anesthesia failure → fine-voxel CBCT (≥2 planes); MRI only for specific soft-tissue questions	Use Gow-Gates or Vazirani–Akinosi plus supplementary infiltrations; avoid instrumentation between limbs; plan shorter implants/altered trajectory; plan conservative osteotomy; hemostasis readiness	Langlais [4]; Naitoh [21]; Göller Bulut [22]; Wadhwani et al. [10]; Asghar et al. [3]; von Arx [12]
Retromolar canal/foramen (RMC)	Hemorrhage; neurosensory disturbance	Accessory neurovascular bundle in the retromolar triangle connecting to MC or crest	PR low sensitivity; PR suspicion or surgery near retromolar area → CBCT to map canal/foramen; MRI rarely needed	Supplemental infiltration in retromolar region; modify flap to avoid foramen; avoid crossing RMC with implants/osteotomy; pre-emptive hemostasis; use piezosurgery if needed	von Arx [12]; Sisman [15]; Shen [16]; Han and Park [23]
Anterior loop (AL)	Mental nerve injury in premolar implant placement	Anterior extension of the IAN beyond the mental foramen	PR unreliable; CBCT to measure loop length and orientation (≥1–3 mm) in ≥2 planes; MRI only for soft-tissue pathway	Individualize safety margin based on CBCT (≥1–3 mm beyond loop); adjust implant length and angulation; plan incision posterior to mental foramen; consider guided surgery; caution with infiltration near MeF	Uchida [18]; Rosa [24]; Khorshidi et al. [25]
High-positioned mandibular canal (HMC)	IAN injury; limited bone height	Short distance between MC and alveolar crest	If PR suggests reduced height or planned split/implant surgery → CBCT to measure MC–crest clearance; treat ≤4–5 mm as high risk	Choose shorter implants or alternative site; modify osteotomy trajectory; consider guided surgery; avoid vertical crest cuts; monitor sensation	Heasman [26]; Polland [27]; Kim [28]; Yu [2]
Accessory mental foramen/accessory exits (AMF/LF)	Accessory nerve injury; bleeding; anesthesia failure	Presence of accessory mental or lateral foramen with additional neurovascular bundle	Plan lateral cortical drilling in premolar region or suspicious PR → CBCT to locate AF; MRI rarely needed	Localize AF on CBCT; reposition incision or fixation to avoid channel; targeted anesthesia away from accessory exits; consider guided surgery; counsel patient on paresthesia risk	Mostafavi [19]; Varvara [29]; Krasny [11]; Öçbe & Borahan [7]

**Table 3:** Embryological Origins of MC and IAN Variants.

Variant	Origin	Clinical Implication	References
BMC, TMC	Incomplete fusion of IAN nerve bundles	Anesthesia failure, nerve injury	Rodríguez-Vázquez et al. [33]; Chávez-Lomeli et al. [35]
RMC	Persistent secondary nerve branches	Bleeding, neuropathy	Lipski et al. [38]; Rodríguez-Vázquez et al. [33]
AF (AMF, LF)	Persistent embryonic vascular channels	Unexpected bleeding, incomplete anesthesia	Murlimanju et al. [38]; Lipski et al. [37]
High MC	Differential mandibular growth	Surgical complications, implant risk	Wyganowska-Świątkowska and Przystańska [34]

**Table 4:** Imaging modalities for MC variants—roles, performance, and limitations (framework summary).

Imaging Modality	Detection Sensitivity	Soft-Tissue Detail	Radiation Exposure	Clinical Applications	Limitations	References
PR	Low for bony variants; susceptible to superimposition and projection geometry	None	Minimal	Initial screening; baseline orientation of dentition and MeF region	Poor spatial resolution; superimposition; cannot confirm small/accessory canals; 2D only	Langlais [4]; Sanchis [5]
CBCT	High for bony canal variants when ≥2-plane confirmation and appropriate voxel size are used; definition- and voxel-dependent	Limited (osseous detail only)	Moderate (apply ALADA principles)	Implant planning; third-molar/ramus surgery; variant mapping (BMC/RMC/AMF); MC–crest clearance; AL assessment	Does not visualize nerve tissue directly; artifacts at very low voxels/metal; targeted cohorts can bias apparent frequencies	Naitoh [21]; Göller Bulut [22]; Asghar [3]
MRI (3T)	Soft-tissue tract visualization (not directly comparable to CBCT “prevalence” of bony canals)	Excellent for neurovascular bundles and perineural pathology	None	Selective use for soft-tissue questions when results would change management (complex branching, perineural/inflammatory pathology)	Cost/availability; motion and metal susceptibility; limited standardization across centers	Krasny [11]; Öçbe [7]
MRI-DTI	Experimental; tractography feasibility in selected cohorts	Excellent (directional information)	None	Advanced nerve pathway mapping in research or highly selected clinical scenarios	Requires further validation; longer acquisition; artifacts and expertise requirements	Kotaki [20]

Notes: Qualitative performance is modality- and threshold-dependent. Percentages are reported per study in Table 1; MRI values are not comparable to CBCT canal prevalence.

**Table 5:** Domain-specific adaptations by variant (anesthesia, implantology, third-molar, osteotomy).

Variant	Anesthesia Adaptation	Implantology/Third-Molar/Osteotomy Adaptation	Imaging & Trigger/Key References
Bifid/Trifid mandibular canal (BMC/TMC)	Prefer Gow-Gates or Vazirani–Akinosi; add buccal/lingual infiltrations; target accessory foramina	Use shorter implant or altered trajectory/site; avoid instrumentation between canal limbs; conservative ostectomy/flap; hemostasis readiness	PR often equivocal → CBCT to map both limbs; MRI only for specific neurovascular questions; revise plan if canal within planned corridor von Arx et al. [12]; Asghar et al. [3]; Wadhwani et al. [10]
Retromolar canal/foramen (RMC)	Supplemental infiltration in retromolar area or alternative block	Avoid crossing RMC with implants/osteotomy/screws; modify flap to spare foramen; vessel control; consider piezosurgery	PR low sensitivity → CBCT to confirm canal/foramen; revise plan if canal intersects surgical path von Arx et al. [12]; Shen et al. [16]; Han & Park [23]
Anterior loop (AL)	Adjust infiltration/incisions near mental foramen; avoid injuring loop	Keep entry/exit corridor posterior to MeF by loop length + buffer; use shorter fixture or adjust angulation; consider guided surgery	PR unreliable → CBCT to estimate loop length/orientation; posteriorize plan or choose shorter implant if uncertain Uchida et al. [18]; Rosa et al. [24]; Khorshidi et al. [25]
High-positioned mandibular canal (HMC)	Consider supplemental infiltrations if block efficacy uncertain	Shorter implant; alter trajectory/site; consider coronectomy for M3 when close; guided surgery; avoid vertical crest cuts	CBCT to measure crest/apex–MC distances; if clearance ≤4–5 mm, shorten implant or change site Heasman [26]; Kim et al. [28]; Yu et al. [2]
Accessory mental foramen/accessory exits (AMF/LF)	Targeted infiltration away from accessory exits	Re-route incisions; avoid screws/fixtures crossing accessory channels; consider guided approach	PR often misses → CBCT mapping; revise plan if accessory exit lies in surgical corridor; MRI rarely required Varvara et al. [29]; Mostafavi et al. [19]; Krasny et al. [11]

**Table 6:** Summary of Future Directions for MC and IAN Management.

Future Direction	Objectives	Clinical Impact	Implementation Strategies
Variant Registries & Big Data	Global MC variant prevalence mapping; outcome correlation	Improved risk stratification, surgical planning	International standardized registry protocols
MRI Tractography Validation	Anatomical validation of MRI nerve tractography with dissections	Precise preoperative nerve mapping	Cadaveric MRI–anatomy correlation studies
AR & Surgical Navigation	Real-time MC visualization during mandibular surgery	Improved intraoperative precision, reduced injuries	Development and validation of AR navigation systems
Educational Reform	Curriculum integration of anatomical variant clinical significance	Enhanced trainee preparedness, proactive mindset	Textbook updates, case-based learning, 3D models

## Data Availability

No new data were created or analyzed in this study. Data sharing is not applicable to this article.

## Supplementary Materials

The following supporting information can be downloaded at: https://www.mdpi.com/article/10.3390/biomedicines13112760/s1, Table S1: Complete database search strategies (PubMed and Scopus). Exact query strings, limits, and search date. No filters beyond those listed were applied.

## Abbreviations

AF	Accessory foramen
AL	Anterior loop
ALADA	As Low As Diagnostically Acceptable
ALARA	As Low As Reasonably Achievable
AMF	Accessory mental foramen
BMC	Bifid mandibular canal
CBCT	Cone-beam computed tomography
DESS	Double-echo steady-state (MRI sequence)
DTI	Diffusion tensor imaging
FOV	Field of view
HMC	High-positioned mandibular canal
IAA	Inferior alveolar artery
IAN	Inferior alveolar nerve
IANB	Inferior alveolar nerve block
ICC	Intraclass correlation coefficient
κ	Cohen’s/Fleiss’ kappa
kVp/mA	Kilovolt peak/Milliampere (X-ray exposure parameters)
LCF	Landfald Clinical Framework
LF	Lingual foramen
MC	Mandibular canal
MeF	Mental foramen
MF	Mandibular foramen
MRI	Magnetic resonance imaging
M3	Mandibular third molar
NV	Neurovascular
PR	Panoramic radiography
RMC	Retromolar canal
TIRM	Turbo inversion recovery magnitude (MRI sequence)
TMC	Trifid mandibular canal

## References

[1] Juodzbalys G., Wang H.L. (2010). Identification of the mandibular vital structures: Practical clinical applications of anatomy and radiological examination methods. J. Oral Maxillofac. Res..

[2] Yu S.K., Lee M.H., Jeon Y.H., Chung Y.Y., Kim H.J. (2016). Anatomical configuration of the inferior alveolar neurovascular bundle: A histomorphometric analysis. Surg. Radiol. Anat..

[3] Asghar A., Priya A., Ravi K.S., Iwanaga J., Tubbs R.S., Naaz S., Panchal P. (2023). An evaluation of mandibular canal variations: A systematic review and meta-analysis. Anat. Sci. Int..

[4] Langlais R.P., Broadus R., Glass B.J. (1985). Bifid mandibular canals in panoramic radiographs. J. Am. Dent. Assoc..

[5] Sanchis J.M., Peñarrocha M., Soler F. (2003). Bifid mandibular canal. J. Oral Maxillofac. Surg..

[6] Elnadoury E.A., Gaweesh Y.S.E., Abu El Sadat S.M., Anwar S.K. (2022). Prevalence of bifid and trifid mandibular canals with unusual patterns of nerve branching using cone beam computed tomography. Odontology.

[7] Öçbe M., Borahan M.O. (2024). Identifying the Anatomical Variations of the Inferior Alveolar Nerve with Magnetic Resonance Imaging. Imaging Niger. J. Clin. Pract..

[8] Dos Santos Oliveira R., Maria Gomes Oliveira A., Cintra Junqueira J.L., Kühl Panzarella F. (2018). Association between the Anatomy of the Mandibular Canal and Facial Types: A Cone-Beam Computed Tomography Analysis. Int. J. Dent..

[9] Altındağ A., Yalın H., Yüksel İ.B. (2025). Pattern diversity and prevalence of bifid mandibular canal: A CBCT-based cross-sectional study: Evaluation of Bifid Mandibular Canal via CBCT. Oral Maxillofac. Surg..

[10] Wadhwani P., Mathur R.M., Kohli M., Sahu R. (2008). Mandibular canal variant: A case report. J. Oral Pathol. Med..

[11] Krasny A., Krasny N., Prescher A. (2012). Anatomic variations of neural canal structures of the mandible observed by 3-tesla magnetic resonance imaging. J. Comput. Assist. Tomogr..

[12] von Arx T., Hänni A., Sendi P., Buser D., Bornstein M.M. (2011). Radiographic study of the mandibular retromolar canal: An anatomic structure with clinical importance. J. Endod..

[13] Page M.J., McKenzie J.E., Bossuyt P.M., Boutron I., Hoffmann T.C., Mulrow C.D., Shamseer L., Tetzlaff J.M., Akl E.A., Brennan S.E. (2021). The PRISMA 2020 statement: An updated guideline for reporting systematic reviews. BMJ.

[14] Kuribayashi A., Watanabe H., Imaizumi A., Tantanapornkul W., Katakami K., Kurabayashi T. (2010). Bifid mandibular canals: Cone beam computed tomography evaluation. Dentomaxillofac. Radiol..

[15] Sisman Y., Ercan-Sekerci A., Payveren-Arikan M., Sahman H. (2015). Diagnostic accuracy of cone-beam CT compared with panoramic images in predicting retromolar canal during extraction of impacted mandibular third molars. Med. Oral Patol. Oral Cir. Bucal.

[16] Shen Y.W., Chang W.C., Huang H.L., Tsai M.T., Fuh L.J., Hsu J.T. (2021). Assessment of the Retromolar Canal in Taiwan Subpopulation: A Cross-Sectional Cone-Beam Computed Tomography Study in a Medical Center. Tomography.

[17] Nascimento E.H.L.D., Pontual M.L.d.A., Pontual A.d.A., Perez D.E.d.C., Figueiroa J.N., Frazão M.A.G., Ramos-Perez F.M.d.M. (2016). Assessment of the anterior loop of the mandibular canal: A study using cone-beam computed tomography. Imaging Sci. Dent..

[18] Uchida Y., Yamashita Y., Goto M., Hanihara T. (2007). Measurement of anterior loop length for the mandibular canal and diameter of the mandibular incisive canal to avoid nerve damage when installing endosseous implants in the interforaminal region. J. Oral Maxillofac. Surg..

[19] Mostafavi M., Zarch S.H.H., Eshghpour M., Khodadadzadeh P. (2024). Prevalence of accessory mental foramen and lateral lingual foramen using cone beam computed tomography: A single-center cross-sectional study. Oral Maxillofac. Surg..

[20] Kotaki S., Sakamoto J., Kretapirom K., Supak N., Sumi Y., Kurabayashi T. (2016). Diffusion tensor imaging of the inferior alveolar nerve using 3T MRI: A study for quantitative evaluation and fibre tracking. Dentomaxillofac. Radiol..

[21] Naitoh M., Hiraiwa Y., Aimiya H., Ariji E. (2009). Observation of bifid mandibular canal using cone-beam computerized tomography. Int. J. Oral Maxillofac. Implants.

[22] Göller Bulut D., Kartal Yalçın G., Tanrıseven Z., Taşkın B., Aydın B. (2024). Prevalence and topography of bifid and trifid mandibular canal in Turkish Western Anatolia Population: Evaluation of the inferior alveolar canal with CBCT. Surg. Radiol. Anat..

[23] Han S.S., Park C.S. (2013). Cone beam CT findings of retromolar canals: Report of cases and literature review. Imaging Sci. Dent..

[24] Rosa M.B., Sotto-Maior B.S., Machado Vde C., Francischone C.E. (2013). Retrospective study of the anterior loop of the inferior alveolar nerve and the incisive canal using cone beam computed tomography. Int. J. Oral Maxillofac. Implants.

[25] Khorshidi H., Raoofi S., Ghapanchi J., Shahidi S., Paknahad M. (2017). Cone Beam Computed Tomographic Analysis of the Course and Position of Mandibular Canal. J. Maxillofac. Oral Surg..

[26] Heasman P.A. (1988). Variation in the position of the inferior dental canal and its significance to restorative dentistry. J. Dent..

[27] Polland K.E., Munro S., Reford G., Lockhart A., Logan G., Brocklebank L., McDonald S.W. (2001). The mandibular canal of the edentulous jaw. Clin. Anat..

[28] Kim S.T., Hu K.S., Song W.C., Kang M.K., Park H.D., Kim H.J. (2009). Location of the mandibular canal and the topography of its neurovascular structures. J. Craniofac. Surg..

[29] Varvara G., Feragalli B., Turkyilmaz I., D’Alonzo A., Rinaldi F., Bianchi S., Piattelli M., Macchiarelli G., Bernardi S. (2022). Prevalence and Characteristics of Accessory Mandibular Canals: A Cone-Beam Computed Tomography Study in a European Adult Population. Diagnostics.

[30] Landfald I.C., Adamek J., Kumar Y.A.S., Olewnik Ł., Coleman J., Labetowicz P. (2025). Anatomy Reimagined: The Landfald Classification as a Transformative Surgical and Radiological Guide to Facial Artery Variants. Ann. Anat.-Anat. Anz..

[31] Landfald I.C., Vazquez T., Okoń A., Olewnik Ł. (2025). Temporalis muscle flap in craniofacial reconstruction: Anatomy, techniques, outcomes, and innovations. Front. Surg..

[32] Okoń A., Landfald I.C., Olewnik Ł. (2025). The Deep Head of the Masseter Muscle: A Classification-Based Anatomical and Surgical Framework. Biomedicines.

[33] Rodríguez-Vázquez J.F., Mérida-Velasco J.R., Mérida-Velasco J.A., Sánchez-Montesinos I., Espín-Ferra J., Jiménez-Collado J. (1997). Development of Meckel’s cartilage in the symphyseal region in man. Anat. Rec..

[34] Wyganowska-Świątkowska M., Przystańska A. (2011). The Meckel’s cartilage in human embryonic and early fetal periods. Anat. Sci. Int..

[35] Chávez-Lomeli M.E., Mansilla Lory J., Pompa J.A., Kjaer I. (1996). The human mandibular canal arises from three separate canals innervating different tooth groups. J. Dent. Res..

[36] Iwanaga J., Takeshita Y., Matsushita Y., Hur M.S., Ibaragi S., Tubbs R.S. (2022). What are the retromolar and bifid/trifid mandibular canals as seen on cone-beam computed tomography? Revisiting classic gross anatomy of the inferior alveolar nerve and correcting terminology. Surg. Radiol. Anat..

[37] Lipski M., Tomaszewska I.M., Lipska W., Lis G.J., Tomaszewski K.A. (2013). The mandible and its foramen: Anatomy, anthropology, embryology and resulting clinical implications. Folia Morphol..

[38] Murlimanju B.V., Latha V., Prameela M.D., Ashraf C.M. (2011). Accessory mandibular foramina: Prevalence, embryological basis and surgical implications. J. Clin. Diagn. Res..

[39] Vranckx M., Geerinckx H., Gaêta-Araujo H., Leite A.F., Politis C., Jacobs R. (2022). Do anatomical variations of the mandibular canal pose an increased risk of inferior alveolar nerve injury after third molar removal?. Clin. Oral Investig..

[40] Muinelo-Lorenzo J., Fernández-Alonso A., Smyth-Chamosa E., Suárez-Quintanilla J.A., Varela-Mallou J., Suárez-Cunqueiro M.M. (2017). Predictive factors of the dimensions and location of mental foramen using cone beam computed tomography. PLoS ONE.

[41] Claeys V., Wackens G. (2005). Bifid mandibular canal: Literature review and case report. Dentomaxillofac. Radiol..

[42] Rouas P., Nancy J., Bar D. (2007). Identification of double mandibular canals: Literature review and three case reports with CTscans and cone beam, C.T. Dentomaxillofac. Radiol..

[43] He S.-X., Ma C., Yuan Z.-Y., Xu T.-F., Xie Q.-T., Wang Y.-X., Huang X.-P. (2024). Feasibility of augmented reality using dental arch-based registration applied to navigation in mandibular distraction osteogenesis: A phantom experiment. BMC Oral Health.

[44] Puleio F., Tosco V., Pirri R., Simeone M., Monterubbianesi R., Giudice G.L., Giudice R.L. (2024). Augmented Reality in Dentistry: Enhancing Precision in Clinical Procedures–A Systematic Review. Clin. Pract..

