# Supplementary material for: Imaging-Based Clinical Management of Mandibular Canal Variants: PR–CBCT–Selective MRI
Source: Biomedicines. 2025 Nov 12;13(11):2760. doi: 10.3390/biomedicines13112760 (PMC12650482; doi:10.3390/biomedicines13112760)
Supplement: Supplementary file 1 [file biomedicines-13-02760-s001.zip › biomedicines-3897839-supplementary.pdf]

Table S1. Complete database search strategies (PubMed & Scopus). Exact query strings, limits, and search date. No filters beyond those listed were applied.

Database (platform)	Date last searched	Coverage / limits	Full query (exact, copy-paste)	Notes
**PubMed (NCBI)**	October 06, 2025	2000–2025; English; Humans; Journal Article/Review	(("Mandibular Canal"[Title/Abstract] OR "Inferior Alveolar Nerve"[Title/Abstract] OR "bifid mandibular canal"[Title/Abstract] OR "trifid mandibular canal"[Title/Abstract] OR "retromolar canal"[Title/Abstract] OR "retromolar foramen"[Title/Abstract] OR "anterior loop"[Title/Abstract] OR "accessory mental foramen"[Title/Abstract] OR "mental foramen"[Title/Abstract]) OR ("Mandibular Canal"[MeSH Terms] OR "Alveolar Nerve, Inferior"[MeSH Terms])) AND ("cone-beam computed tomography"[Title/Abstract] OR CBCT[Title/Abstract] OR "panoramic radiography"[Title/Abstract] OR orthopantomogram[Title/Abstract] OR orthopantomography[Title/Abstract] OR OPG[Title/Abstract] OR "magnetic resonance imaging"[Title/Abstract] OR MRI[Title/Abstract]) AND english[lang] AND ("2000/01/01"[dp] : "2025/10/30"[dp]) AND (Review[Publication Type] OR Journal Article[Publication	Included MeSH for MC/IAC; date range via [dp].

			Type]) AND Humans[MeSH Terms]	
**Scopus (Elsevier)**	October 30, 2025	PUBYEAR > 1999; English; Article/Review	TITLE-ABS-KEY("mandibular canal" OR "inferior alveolar nerve" OR "bifid mandibular canal" OR "trifid mandibular canal" OR "retromolar canal" OR "retromolar foramen" OR "anterior loop" OR "accessory mental foramen" OR "mental foramen") AND TITLE-ABS-KEY("cone-beam computed tomography" OR CBCT OR "panoramic radiography" OR orthopantomogram OR orthopantomography OR OPG OR "magnetic resonance imaging" OR MRI) AND DOCTYPE(ar OR re) AND LANGUAGE(english) AND PUBYEAR > 1999	TITLE-ABS-KEY used; DOCTYPE/LANGUAGE/PUBYEAR limits.
